# Long non-coding RNA H19 expression and functional polymorphism rs217727 are linked to increased ischemic stroke risk

**DOI:** 10.1186/s12883-021-02081-3

**Published:** 2021-02-04

**Authors:** Mohadese Rezaei, Mohammad Javad Mokhtari, Mahnaz Bayat, Anahid Safari, Mehdi Dianatpuor, Reza Tabrizi, Tahereh Asadabadi, Afshin Borhani-Haghighi

**Affiliations:** 1 Department of Biology, Zarghan Branch, Islamic Azad University, 4341617184, Zarghan, Iran; 2 Clinical Neurology Research Center, Shiraz University of Medical Sciences, Shiraz, Iran; 3 Stem Cells Technology Research Center, Shiraz University of Medical Sciences, Shiraz, Iran; 4 Noncommunicable Diseases Research Center, Fasa University of Medical Sciences, Fasa, Iran

**Keywords:** Long non coding- RNA, Ischemic stroke, Diagnosis, Biomarker, Gene polymorphism

## Abstract

**Background:**

Efforts to identify potential biomarkers for the diagnosis of ischemic stroke (IS) are valuable. The H19 gene plays a functional role in increasing the prevalence of IS risk factors. We evaluated the correlation between H19 rs217727 polymorphism and the expression level of H19 lncRNA with susceptibility to IS among the Iranian population.

**Methods:**

Blood samples were collected from IS patients (*n* = 114) and controls (*n* = 114). We concentrated on the expression pattern of H19 at different time points (i.e., 0–24, 24–48, and 48–72 h after stroke). The tetra-amplification refractory mutation system-polymerase chain reaction (T-ARMS-PCR) method was applied for DNA genotyping. We used the quantitative real-time PCR to evaluate H19 expression levels. We used the receiver operating characteristic (ROC) curve to evaluate the diagnosis and prognosis of IS.

**Results:**

The rs217727polymorphism of H19 was related with IS susceptibility in the co-dominant (OR = 2.92, 95% CI = 0.91–10.92, *P* = 0.04) and recessive models (OR = 2.80, 95% CI = 0.96–8.15, *P* = 0.04). H19 expression was significantly upregulated in IS and remained high for 72 h after stroke. ROC curves showed that H19 expression within the first 24 h from stroke onset might serve as a biomarker for the early diagnosis of IS with 79.49% sensitivity and 80.00% specificity. H19 expression in small vessel occlusion (SVO) and large-artery atherosclerosis (LAA) patients were 3.74 and 3.34 times higher than the undetermined (UD) subtype, respectively [OR = 3.74 95% CL (1.14–12.27) *P* = 0.030 and OR = 3.34 95% CL (1.13–9.85) *P* = 0.029].

**Conclusion:**

The rs217727 polymorphism of the H19 is correlated with IS susceptibility, and H19 expression levels were higher in SVO and LAA patients. The upregulation of H19 may be considered as a diagnostic biomarker in IS among the Iranian population, but it cannot serve as a useful prognostic marker.

## Background

Stroke is one of the leading causes of death globally (accounting for 5.5 million deaths annually). The high proportion of stroke patients are young adults in Iran, with a higher mortality rate compared to Western countries [1]. Ischemic stroke (IS) accounts for 75% of all stroke cases. IS patients often suffer from disability and loss of productivity, which can cause a heavy socioeconomic burden [2]. Based on different molecular studies, the clarification of genetic biology, and epigenetic can facilitate the prediction and diagnosis of severe strokes [3].

Long non-coding RNAs (LncRNAs) are RNAs that have more than 200 nucleotides and lack protein-coding capacity. Documents have increasingly revealed that dysregulated lncRNA expression levels can lead to various human diseases [4, 5] . lncRNAs are highly expressed in neural cells, and there is a strong likelihood that they are involved in brain dysfunction [6–8]. Recent research works have shown that the expression of selected lncRNA changes over time after IS is detected in the blood of patients [9–11]. H19 lncRNA is the primary transcript of H19 locus; after birth, the abnormal expression of lncRNA H19 was presented as a potential diagnostic biomarker in different cancers [12]. Additionally, LncRNA H19 is typically re-expressed in atherosclerotic plaque [12], and it can be activated under hypoxic conditions [13].

LncRNAs H19 has been revealed to affect the pathophysiology of IS by promoting neuroinflammation [14], preventing neurogenesis [15], and increasing the risk of atherosclerosis [16]. Notably, previous studies have suggested that the genetic polymorphisms on lncRNA influence lncRNA expression and, thus, affect disease susceptibility [8, 17]. Gao et al. (2014) reported that rs217727 polymorphism in the H19 gene was correlated with an increased risk of coronary artery disease [18]. Moreover, Ghaedi et al. demonstrated an association between H19 rs217727 polymorphism with type 2 diabetes susceptibility [19]. Coronary artery disease and diabetes are two main risk factors for IS [20, 21]. Zhu et al. noted that H19 rs217727 gene polymorphism can serve as a marker for IS in the northern Chinese population [22]. On the other hand, Haung et al. (2019) reported that the rs217727 polymorphism was not correlated with IS in the southern Chinese population [23]. Perhaps the main reason for this inconsistency is that the genetic heterogeneity in different areas. Thus, more studies are needed to explain the association between rs217727 polymorphism and IS risk in the populations of different countries.

Previous studies suggested that rs217727 polymorphism is related to the pathogenesis of breast cancer, oral squamous cell carcinoma, and type 2 diabetes susceptibility in the Iranian population [19, 24, 25]. Research on the role of lncRNAs in IS pathogenesis is just beginning, and we have performed this study to assess the association between lncRNA H19 expression and its rs217727 polymorphism with susceptibility to IS in the Iranian population. Especially given the importance of the alteration in lncRNA expression after IS over time, we compared the change in the expression level of lncRNA H19 in three different times (0–24, 24–48, 48–72 h after the onset of symptoms. The importance of this study becomes more apparent due to the high prevalence of IS in the Iranian population [26]. Therefore, our effort to identify a potential diagnostic marker for IS is valuable. In the current study, the diagnostic potential of lncRNA H19 level as a biomarker in IS patients was evaluated by ROC curve analysis and were compared at different times after stroke.

## Methods

### Study subjects

This case-control study was conducted from August 2018 to August 2019 at Namazi Hospital in Shiraz, a high-volume referral center in the south of Iran. According to the Recognition of Stroke in the Emergency Room (ROSIER) scale, IS was screened as a focal neurological deficit that persisted beyond 24 h in surviving patients. Then, radiological confirmation was performed via a brain CT or MRI. IS was defined according to international guidelines [27]. Patients with IS aged above 18 years who filled the informed consent were included in the study. Exclusion criteria were primary intracranial hemorrhage, arterial dissection, cerebral vasculitis, cerebral venous thrombosis, hypoperfusion syndromes, iatrogenic stroke, thrombosed intracranial aneurysm, meningitis, thrombotic thrombocytopenic purpura-hemolytic uremic syndrome, Moyamoya disease, cerebral vasoconstriction syndrome, and malignancy. Control participants were a representative sample of the Shiraz population who were randomly selected from neighbor controls at the nearest from the case’s place, sex, and age frequency matched with cases. The examined subjects were aged 32–90 years. Controls with a history of stroke, transient ischemic attack, illness or disorder of the brain, or malignancy were excluded.

The National Institutes of Health Stroke Scale (NIHSS) score was assessed upon admission. Higher scores indicated increased severity. The outcomes were obtained 3 months after admission according to the modified Rankin Scale (mRS) blinded to H19 levels. Ethics approval for this research was obtained by the local ethics committee of Kazeroon Branch, Islamic Azad University with the number 97 01 94-19386.

### Collection of blood samples

After obtaining informed consent, peripheral venous blood samples were collected from IS patients 0–24 (*n* = 39), 24–48 (*n* = 38), and 48–72 (*n* = 37) hours after the onset of symptoms, and control subjects in ethylene diamine tetra acetic acid-coated (EDTA) tubes as an anticoagulant.

### SNP genotyping and measurement of the lncRNA H19 levels

DNA was isolated from the blood samples using the DNA Extraction Kit (Favorgen, Taiwan).

To evaluate the purity and concentration of the isolated DNA, a photo nanometer was applied at 230, 260, and 280 nm ed. Polymorphism has been evaluated by a tetra-amplification refractory mutation system-polymerase chain reaction (T-ARMS-PCR) as described previously by Ghapanchi et al. (2020) [24]. Using the Tera-ARMS PCR method in genomic research allows for rapid SNP identification in a reliable (accuracy of 99.9%), and low-cost manner [28]. Sanger sequencing was done on approximately 20% of the random samples and demonstrated no genotyping error.

Total RNA was extracted from fresh blood samples using a total RNA extraction kit (Favorgen, Taiwan) following the manufacturer’s instructions. To remove DNA contamination, the isolated RNA was treated with DNase I enzyme. RNA samples were used for cDNA synthesis using the cDNA synthesis Kit (Yektatajhiz, Iran). The relative abundance of the H19 level was measured by quantitative real-time PCR using RealQ Plus 2x Master Mix Green Low ROX™ (Ampliqon, Denmark). Here, we used the Quantstudio 3 Real-Time PCR System (Applied Biosystems, Foster City, USA) with the thermal-cycling settings of 10 min at 95 °C (1 repeat) accompanied by 40 cycles for 15 s at 95 °C and 1 min at 60 °C. Every complete amplification phase was accompanied by a melting phase, for 15 s at 95°C, 30s at 60°C, and 15 s at 95°C. The following primers were used: for the H19 5′- GCAGACAGTACAGCATCCA − 3′ (forward) and 5′-CTCCTGAGAGCTCATTCACTC − 3′ (reverse); for the TATA-Binding Protein (TBP, reference gene), 5′-CCCGAAACGCCGAATATAATC-3′ (forward) and 5′-TCTGGACTGTTCTTCACTCTTG-3′ (reverse).

To normalize the target gene expression, the *TBP* gene was selected. 2^-ΔCT^ was used to calculate the H19 comparative expression level for each individual [29].

### Statistical analysis

We evaluated the Hardy-Weinberg equilibrium (HWE) by using a chi-squared (χ^2^) test to compare the expected genotype frequencies with the observed ones among the controls. Further, used were unconditional logistic regression analyses to test each SNP’s relationship with case/control status under different genetic models, such as dominant, co-dominant, recessive, and over-dominant. Odds ratios (ORs) and their corresponding 95% confidence intervals (95% CI) were scored to assess the strength of association between the risk of IS and H19 polymorphisms. The relationship of the H19 expression level with main and clinical variables was estimated in multiple logistic regression analyses. The median level of H19 expression was considered as cutoff value for logistic regression. Comparisons were made between categorical data was assessed by using a chi-square test. The differences between numeric variables were evaluated using an independent two-sample t-test. Correlations were analyzed using the Spearman correlation. The ROC curve analysis was done to estimate the diagnostic and prognostic values, and the findings were shown as the area under the curve (AUC). The analyses were done using the version 19.0 of SPSS software and GraphPad Prism 5.0. A *P*-value of < 0.05 was regarded as statistically significant.

## Results

### Clinical characteristics of IS patients and controls

There was no statistically significant difference between the IS patients and the healthy control patients with respect to age, sex, and BMI (*P* > 0.05, Table 1). However, IS patients’ levels of hypertension and smoking (*P* < 0.001) diabetes (*P* < 0.003), drinking (*P* < 0.005), and dyslipidemia (*P* < 0.026) were significantly higher than those of the controls (Table 1).

**Table 1:** Clinical characteristics of study subjects

Characteristics	Cases (*n* = 114)	Controls (*n* = 114)	*p*
Male, n (%)	68 (59.64)	68 (59.64)	0.999^a^
Female, n (%)	46 (40.35)	46 (40.35)	
Age (years)	65.88 ± 14.44	65.88 ± 14.44	0.999^b^
BMI (kg/m2)	25.80 ± 5.21	26.21 ± 4.51	0.523
NIHSS at admission			
≤ 6	63 (55.26)	–	
≥ 7	51 (44.73)		
mRS at 3 months at admission			
0–2	31 (29.19%)	–	
3–6	83 (72.80%)		
mRS at 6 months			
0–2	72 (63.15%)	–	
3–6	42 (36.84%)		
Toast of classification cases n (%)			
LAA	34(29.82)		
SVO	26(22.8)		
CE	26(22.8)		
UD	28(24.5)		
Vascular risk factors			
Hypertension, n (%)	65 (57.02)	31 (27.20)	< 0.001^a^
Diabetes, n (%)	35 (30.7)	16 (14.04)	0.003^a^
Smoking, n (%)	34 (29.82)	8 (7.02)	< 0.001^a^
Drinking, n (%)	16 (14.03)	3 (2.63)	0.005^a^
Dyslipidemia	39 (34.21)	24(21.05)	0.026^a^

Data were shown as mean ± standard deviation (SD) or as n (%).^a^Chi-square Test; ^b^Independent two-sample T-test. *P*–value of < 0.05 was regarded as statistically significant

*NIHSS* National institutes of health stroke scale, *LAA* large-artery atherosclerosis, *SVO* small vessel occlusion, *CE* cardio embolism, *UD* undetermined etiology, *BMI* Body Mass Index

### H19 rs217727 polymorphism has a positive association with the risk of IS

We did not find any significant deviations from the Hardy-Weinberg equilibrium for rs217727 polymorphism in the control group. The results confirmed the association between the IS and rs217727polymorphism in Iranian individuals. We found that the C allele of rs217727 was a significant protective factor against IS in the Iranian populations (C vs. T: OR = 0.62, 95% CI: 0.39–0.99, *P* = 0.04). Furthermore, the frequency of TT genotypes of H19 rs217727 was significantly higher in IS patients than in healthy controls (11. 4 vs. 4.38%). Moreover, the TT genotype was associated with a 2.92-fold increase in IS risk in the co-dominant model (OR = 2.92, 95% CI = 0.91–10.92, *P* = 0.04). Also, H19 rs217727 polymorphism was related to a 2.80-fold increase in the risk of IS in the recessive model (TT vs. CC + CT genotypes) (Table 2).

**Table 2:** Allele and genotype frequencies of *H19* rs217727 for IS patients and the control group

Inheritance model	rs217727 Polymorphism	Cases (%)	Controls (%)	OR (95% CI)	*P*
Codominant^a^	Genotype				
	CC	73 (64.03)	82 (71.92)	1	
	CT	28 (24.56)	27 (23.68)	1.16 (0.62–2.15)	0.62
	TT	13 (11.40)	5 (4.38)	2.92 (0.91–10.92)	0.04
Dominant^b^	CC	73 (64.03)	82 (71.92)	1	
	CT + TT	41 (35.96)	32 (28.07)	1.43 (0.82–2.51)	0.20
Recessive^c^	CC + CT	101 (88.59)	109 (95.61)	1	
	TT	13 (11.40)	5 (4.38)	2.80 (0.96–8.15)	0.04
Over-dominant^d^	CC + TT	86 (75.43)	87 (76.31)	1	
	CT	28 (24.56)	27 (23.68)	1.04 (0.57–1.92)	0.87
	Allele				
	T	54 (23.68)	37 (16.22)	1	
	C	174 (76.31)	191 (83.77)	0.62 (0.39–0.99)	0.04

*OR* odd ratio, *CI* confidence interval

^a^Co-dominant; major allele homozygotes vs. heterozygotes

^b^Dominant; major allele homozygotes vs. heterozygotes + minor allele homozygotes

^c^Recessive; major allelehomozygotes + heterozygotes vs. minor allele homozygotes

^d^Over dominant; major allele homozygotes + minor allele homozygotes vs. heterozygotes

### H19 expression was significantly upregulated in the peripheral blood of IS patients

**Fig. 1:**
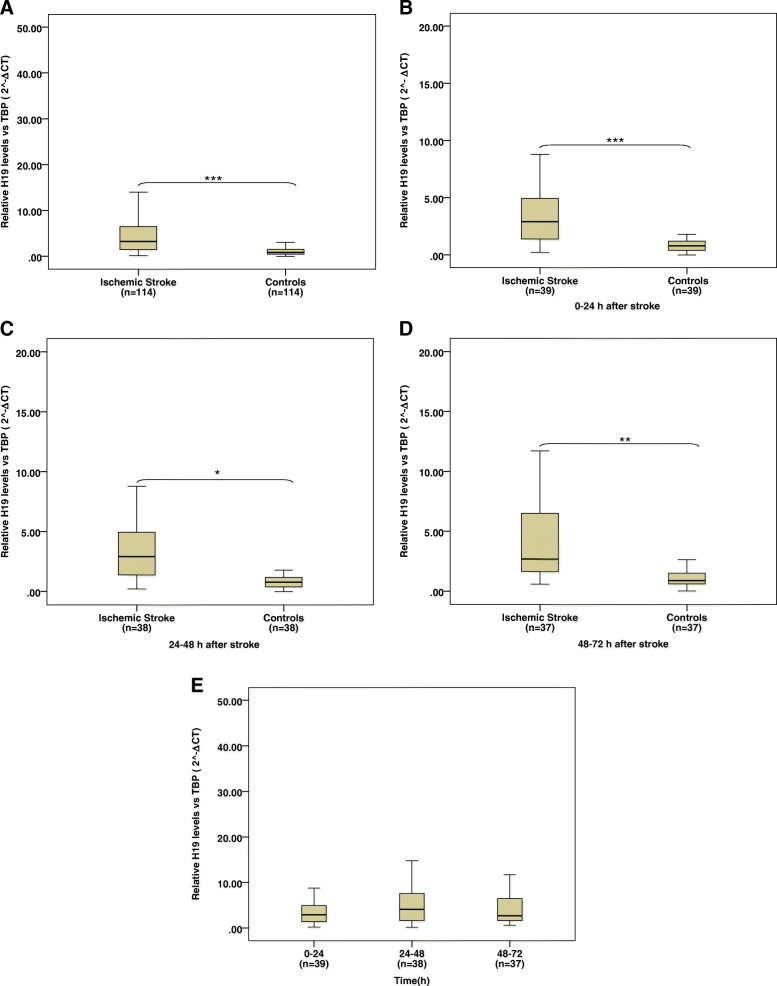
The expression levels of H19 in different time points. H19 expression level in IS was significantly more than those in controls (**a**). H19 levels in IS patients 0–24 (**b**), 24–48 (**c**), 48–72 (**d**) hours after symptom onset were higher than the controls, while no significant differences were found between 3 time points (**e**). Results were expressed as mean ± SE. Significance was set at (*P < 0.05, **P < 0.01, ***P < 0.001)

We found that H19 expression was significantly upregulated in IS cases when compared to controls (Fig. 1a, *P* < 0.001). Further analysis indicated that H19 expression was found more often in IS patients than in the controls 0–24, 24–48, and 48–72 h after the onset of stroke (*P* < 0.001, *P* < 0.05, *P* < 0.01, respectively). An independent t-test was used to compare the relative H19 expression in peripheral blood of IS cases at each time point with age- and sex-matched controls. H19 expression levels in IS patients were compared to levels of controls 0–24 h (4.51 ± 0.88 vs 0.96 ± 0.12), 24–48 h (7.12 ± 1.53 vs 2.73 ± 1.19), and 48–72 h (6.38 ± 1.36 vs 1.84 ± 0.36) after stroke (Fig. 1b-d). After adjusting for potential confounding factors such as sex, age, BMI, diabetes, hyperlipidemia, hypertension, cigarette smoking and alcohol drinking, in the logistic regression model, it was found that H19 expression was significantly associated with ischemic stroke (*P* = 0.001). Interestingly, we observed that upregulated H19 remained high 72 h after a stroke (Fig. 1e).

### Association between clinical variables and H19 expression

Subgroup analyses were performed to define the association between clinical variables and H19 expression (Table 3). We did not find any significant differences regarding other characteristics.

**Table 3:** Association between *H19* levels and clinical characteristics in IS patients

Characteristics	n	Mean ± SD	t	*p*
Gender			–0.118	0.906
Male	68	5.94 + 8.12		
Female	46	6.12 + 8.13		
Age			1.64	0.103
< 70	63	7.03 ± 9.69		
≥ 70	51	4.71 ± 5.33		
NIHSS at admission			0.871	0.162
≤ 6	62	6.12 ± 8.54		
≥7	52	5.87 ± 7.58		
Hypertension			−0.026	0.979
Negative	49	6.03 ± 1.75		
Positive	65	6.07 ± 10.57		
Diabetes			−1.215	0.227
Negative	79	5.44 ± 7.56		
Positive	35	7.47 ± 9.32		
Smoking			−0.810	0.420
Negative	80	6.49 ± 8.27		
Positive	34	5.10 ± 7.89		
Drinking			0.271	0.787
Negative	98	5.97 ± 7.69		
Positive	16	6.57 ± 10.86		
High Density Lipoprotein, mmol/L			0.241	0.810
≤ 49	76	5.92 ± 7.75		
≥ 50	38	6.31 ± 9.01		
Low Density Lipoprotein, mmol/L			−1.89	0.063
≤ 99	67	4.74 ± 5.82		
≥100	47	7.92 ± 10.42		
Triglycerid, mmol/L			−0.883	0.379
≤ 138	67	5.49 ± 6.93		
≥139	47	6.86 ± 9.65		
Total Cholesterol, mmol/L			0.671	0.50
≤ 148	54	6.59 ± 8.82		
≥149	60	5.57 ± 7.54		

Data were shown as mean ± standard deviation (SD) and Independent two-sample T-test

*P*–value of < 0.05 was regarded as statistically significant

*NIHSS* National institutes of health stroke scale

We showed that the H19 expression level was not significantly correlated with clinical characteristics.

### H19 expression was associated with TOAST subtypes of IS

**Fig. 2:**
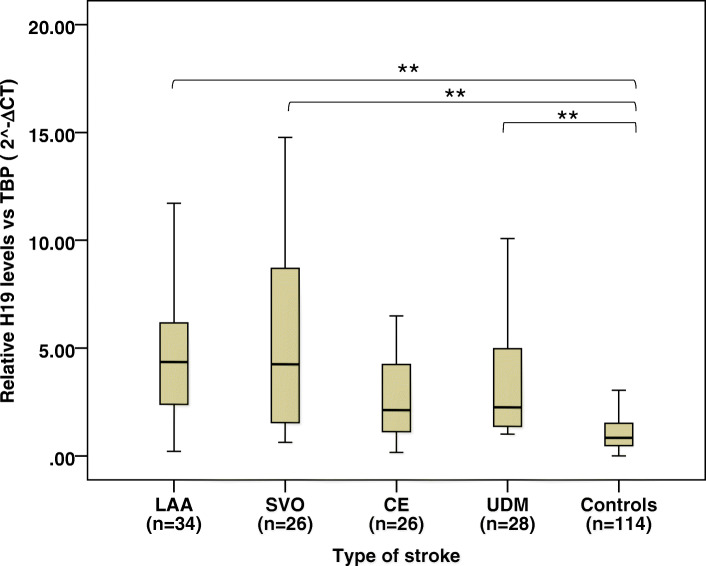
H19 expression was associated with TOAST subtypes of IS patients. H19 levels in LAA, SVO and UD groups were significantly higher than those controls. Data were shown as mean ± SE. Abbreviations: LAA: large-artery atherosclerosis; SVO: small-vessel occlusion; CE: cardioembolism; UD: undetermined

Patients were assigned to four subtypes according to TOAST classification (Table 1). We found a significant elevation in H19 expression in patients with LAA, SVO, and UD when compared to controls (ANOVA, *P* < 0.01). IS patients with CE showed the lowest levels of H19 without a significant difference with healthy controls (Fig. 2) (*P* > 0.05).

Multiple logistic regression analysis was done to detect the association between TOAST subtypes and H19 expression after adjusting for several confounders (Table 4). We showed that H19 expression levels were significantly associated with TOAST subtypes. H19 expression levels in SVO and LAA patients were 3.74 and 3.34 times higher than the UD subtype, respectively [OR = 3.74 95% CL (1.14–12.27) *P* = 0.030 and OR = 3.34 95% CL (1.13–9.85) *P* = 0.029].

**Table 4:** The results of univariate and multiple linear regression analyses

Characteristics	Univariate			Multiplevariate ^a^		
	Beta	95% CI	*P*-Value	Beta	95% CI	*P*-Value
Male sex	−0.29	−3.39, 2.81	0.854	0.16	−3.37, 3.69	0.928
Age (year)	−0.05	−0.15, 0.06	0.379	−0.02	− 0.14, 0.11	0.819
Hypertension	0.88	−2.19, 3.95	0.571	0.06	−3.33, 3.44	0.974
Diabetes mellitus	2.01	−1.27, 5.28	0.227	2.20	−1.48, 5.87	0.238
Dyslipidemia	0.07	−3.13, 3.28	0.964	−1.42	−5.13, 2.18	0.451
Cigarette Smoking	−1.35	−4.68,1.96	0.420	−1.58	−5.34, 2.18	0.406
NIHSS score	0.01	−0.23, 0.24	0.976	0.08	− 0.19, 0.38	0.574
Alcohol use	0.60	−03.78, 4.97	0.787	0.67	−4.17, 5.52	0.783
Type of IS UD	Ref			Ref		
CE	−2.55	−6.97, 1.87	0.255	−1.36	−6.16, 3.45	
SVO	0.43	−3.99, 4.85	0.849	1.28	−3.50, 6.06	
LAA	−0.52	−4.66, 3.62	0.803	−0.17	−4.48, 4.15	
BMI	0.24	−0.05, 0.53	0.103	0.26	−0.09, 0.61	0.141

*P*–value < 0.05 was regarded as statistically significant

*NIHSS* National institutes of health stroke scale, *BMI* Body Mass Index, *LAA* large-artery atherosclerosis, *SVO* small vessel occlusion, *CE* cardio embolism, *UD* undetermined etiology

^a^Multiple linear regression was conducted using enter method with adjustments for the most important clinical and main factors

### H19 expression levels in different genotypes of rs217727 polymorphism in IS patients

**Fig. 3:**
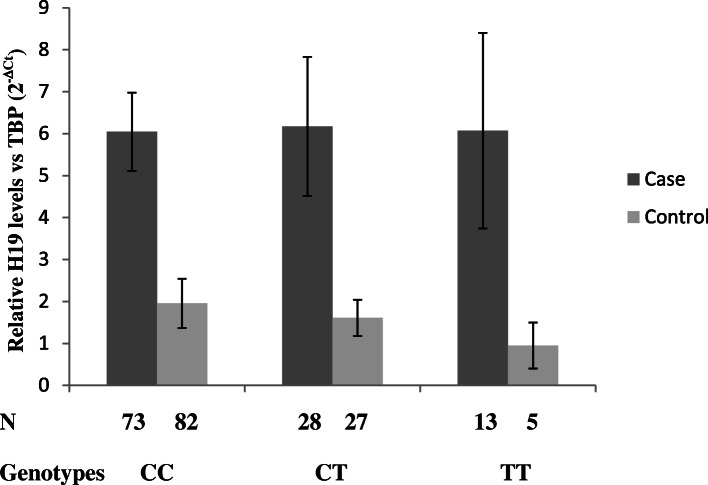
The comparison of relative expression levels of H19 lncRNA between different genotypes of rs217727 polymorphism in cases and controls. Data were shown as mean ± SE. N shows the number of participants with each genotype

Further analysis revealed that there were no significant differences in relative H19 expression levels among the three groups of rs217727 (CC, CT and TT) genotypes in case (*F* = 0.00; df = 2, *P* = 0.999) and control (*F* = 0.14; df = 2, *P* = 0.865) (Fig. 3).

### Diagnostic value of H19 expression level in peripheral blood

**Fig. 4:**
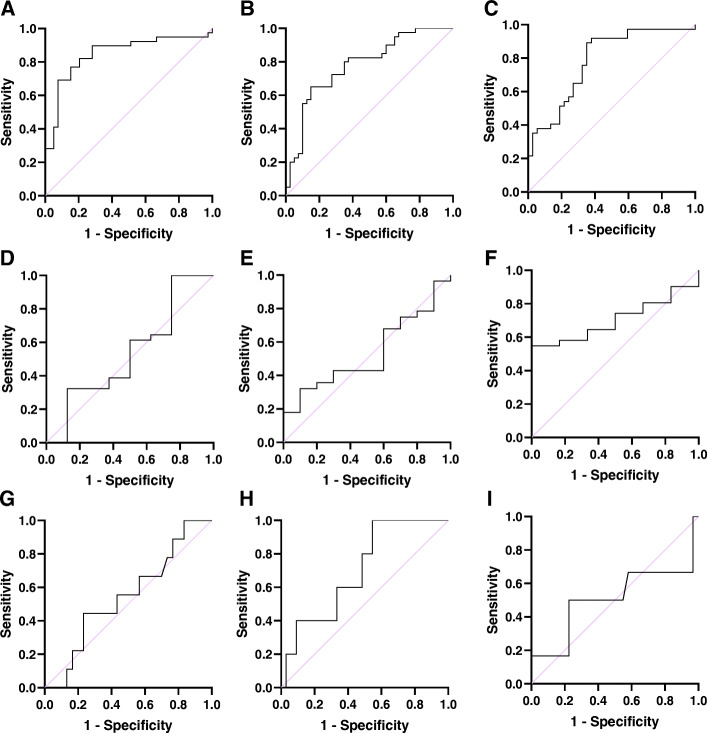
ROC curves. ROC curve analysis showed that H19 was a potential biomarker for IS diagnosis cases from the controls, 0–24 h (**a**), 24–48 h (**b**), 48–72 h (**c**) after IS. H19 for discriminating with patients unfavorable outcomes from those with favorable outcomes, 0–24 h (**d**), 24–48 h (**e**), 48–72 h (**f**) after IS. H19 for discriminating non survivors from survivors, 0–24 h (**g**), 24–48 h (**h**), 48–72 h (**i**) after IS

The ROC curve analysis showed that H19 is a potential biomarker for IS diagnosis cases (Table 5). For the period 0–24 h after IS, AUC was 0.874 ± 0.039 (95% CI, 0.796–0.951; Fig. 4a); the sensitivity and specificity were 79.49%. For the period 24–48 h after IS, AUC was 0.813 ± 0.049 (95% CI, 0.715–0.910; Fig. 4b), and the sensitivity and specificity were 77.5 and 75.00%, respectively. For the period 48–72 h after IS, AUC was 0.788 ± 0.052 (95% CI, 0.685–0.891; Fig. 4c), and the sensitivity and specificity were 75.68 and 67.57%, respectively. Circulating H19 levels within the first 24 h after the onset of a stroke indicated high sensitivity and specificity for the early diagnosis of acute stroke.

**Table 5:** Prediction of diagnostic value, functional outcome and mortality

Parameter	AUC ± SE	95% CI	*P*	Optimal threshold	Se (%)	Sp (%)
Diagnostic value						
Expression H19 for first 24 h interval	0.874 ± 0.039	0.796–0.951	< 0.0001	1.246	79.49%	79.49%
Expression H19 for second 24 h interval	0.813 ± 0.049	0.715–0.910	< 0.0001	1.534	77.5%	75.00%
Expression H19 for 3rd 24 h interval	0.788 ± 0.052	0.685–0.891	< 0.0001	1.614	75.68%	67.57%
Prediction of functional outcome						
Expression H19 in 24 h	0.571 ± 0.065	0.430–0.707	0.278	3.990	58.98%	64.10%
Expression H19 in 48 h	0.589 ± 0.070	0.451–0.727	0.168	2.950	52.50%	87.50%
Expression H19 in 72 h	0.549 ± 0.071	0.409–0.690	0.485	3.615	60.53%	54.05%
Prediction of mortality						
Expression H19 for first 24 h interval	0.566 ± 0.068	0.435–0.698	0.310	2.085	64.10%	48.72%
Expression H19 for second 24 h interval	0.778 ± 0.051	0.678–0.819	< 0.0001	2.170	75.00%	67.50%
Expression H19 for 3rd 24 h interval	0.706 ± 0.061	0.586–0.826	0.002	2.061	70.27%	60.53%

*Abbreviations*: *AUC* Area Under the Curve, *CI* Confidence Interval, *Se* sensitivity, *Sp* specificity *P* < 0.05 was considered statistically significant

Also, to assess whether H19 could be identified as a functional outcome and mortality prediction marker, ROC curve analyses for IS cases at various time points were drawn. ROC curve analysisindicated that the peripheral blood H19 expression level could not be considered as a promising marker for the functional outcome and mortality prediction of IS patients. Results were shown in Table 5 and Fig. 4.

At a 3-month follow-up, 26 cases (22.80%) died and 88 cases (77.19%) survived. Survivors had lower H19 expression levels (7.32 ± 5.77) than the dead patients (10.26 ± 6.75); however, this difference was not significant (*P* = 0.579).

## Discussion

This research showed the effect of H19 gene polymorphism (rs217727) on IS susceptibility through a case-control study on individuals from southwestern Iran. The findings demonstrated that rs217727 polymorphism was significantly associated with the risk of IS in the co-dominant and recessive models. However, it did not correlate with H19 expression levels in the peripheral blood of Iranian patients. In addition, we observed a significant increase in the lncRNA H19 expression levels of IS patients when compared to healthy controls. This result is consistent with previous studies that reported the upregulation of H19 in the peripheral blood of IS patients [14, 23, 30]. We evaluated H19 expression at three different times (0–24 h [*n* = 39], 24–48 h [*n* = 38], and 48–72 h [*n* = 37] after stroke). We demonstrated that lncRNA H19 expression significantly increased in IS patients and remained high for 72 h after a stroke. Xiao et al. reported a high expression level of H19 in 40 IS patients within the first 3 h after a stroke. This finding was in line with the results of Wang et al. and Haung et al., whose results indicated high levels of lncRNA H19 for the first 24 h after IS stroke (in 36 and 64 IS patients, respectively) [14, 23].

The results obtained from in vitro and in vivo experiments showed that H19 was upregulated following ischemic reperfusion injuries in the ischemic penumbra of animals, as well as in the neuroblastoma cell after oxygen-glucose deprivation and reperfusion (OGD/R)-induced injury [30–32]. We have little information about the basic mechanism of lncRNA H19 in IS. Gao et al. (2020) reported that lncRNA H19 could aggravate oxidative stress and apoptosis in I/R tissues and OGD/R-induced cells via a PTEN/Akt signaling pathway, whereas H19 was overexpressed and miR-19a-3p was down-regulated [32]. Recently, Xiao et al. reported neuronal apoptosis (both in vitro and in vivo) was reduced by the inhibition of H19 with siRNA. Also, the inhibition of H19 in ischemic rats significantly decreased brain infarct size, neurological deficit, and neuronal apoptosis [30]. Wang et al. demonstrated that lncRNA H19 caused by activation of autophagy induces cerebral ischemia reperfusion damage [31]. During cerebral ischemia reperfusion, autophagosome acts as a protective mechanism since it can recover the energy and materials by clean up the damaged organelles [33]. However, excessive autophagy activation plays an important role in the induction of apoptosis, necrosis, and the autophagic death of neurons following cerebral I/R [34]. Furthermore, lncRNA H19 has a critical role in atherosclerosis-related pathophysiology and the development of atherosclerosis [35]. In this regard, Haung et al. reported that the expression of acid phosphatase 5 protein is promoted by the H19 gene. This protein contributes to atherosclerosis and increases the risk of IS [16].

In our patients, the levels of upregulated H19 expression remained high until 72 h after the onset of symptoms. It may take more time to recover to normal levels. The maximum recovery in the expression of different lncRNAs after stroke can take up to 7 days [11]. Another important finding was that circulating H19 levels were positively associated with LAA and SVO subtypes. Atherosclerosis is a predictable risk factor for LAA and SVO subtypes [36]. This is consistent with another observation that showed that H19 is correlated with LAA subtypes of atherosclerotic patients [16]. Many investigations have demonstrated that H19 is involved in the native atherosclerosis via different mechanisms, such as cellular proliferation and apoptosis, adipocyte differentiation, inflammatory response, lipid metabolism, and regulation of angiogenesis [35]. Recent studies have shown that H19 might play a critical role in the onset and progression of atherosclerosis [23, 37]. We found that patients with the CE subtype had the lowest levels of H19 expression. This finding was also reported by Huang et al. (2019) [16].

In the present investigation, lncRNA H19 expression was not positively correlated with NIHSS scores. This outcome is contrary to the findings of Xiao et al. (2019), who found a positive correlation between NIHSS scores and H19 expression 3 h after stroke onset [30]. It could be argued that the observed positive association by Xiao et al. was due to blood sampling within the first 3 h. Meanwhile, in our study, blood samples were collected at three different times. The main reason for this discrepancy can be explained by the assumption that there are optimal blood sampling time windows for estimating a positive association between elevated circulating H19 and NIHSS scores.

A valuable method for early screening of IS is developing a high-sensitive noninvasive blood biomarker. Evidence shows that circulating lncRNAs could be considered an IS biomarker [38, 39]. Elevated H19 expression has been reported in various oncological disorders [12, 25]. Therefore, it can be used as a non-specific biomarker for IS. Subjects with known oncological diseases were excluded from the present study. The current study showed that circulating H19 levels within the first 24 h after the onset of a stroke have a sensitivity of 79.4% and a specificity of 80%. Therefore, H19 expression could serve as a potential biomarker for the early diagnosis of acute stroke; after 24 h, its sensitivity and specificity decreased over time. The upregulation of H19 cannot serve as a useful prognostic marker.

We showed that the TT genotype of the rs217727 polymorphism was significantly correlated with increased risk of IS. This is in line with the results of Zhu et al. and Wang et al. [22, 31]. However, the expression levels of H19 in different genotypes of the rs217727 polymorphism did not show a significant difference. Probably, the rs217727 polymorphism affects the lncRNA H19 expression within the first few hours after the onset of IS. Therefore, the current study could not detect this issue, or perhaps mutations changed the translational efficiency, thereby leading to changes in lncRNA H19 structure and expression.

As a shortcoming, H19 expression level was measured in different cases for each of time points (0–24, 24–48, and 48–72 h). It was because some patients admitted more than 24 h after evolution of IS and some were discharged or died before 48 h or 72 h. Nonetheless, this methodology has been used by other studies [40].

In this study, all patients were chosen consecutively from hospitals during the same period with various ethnic groups, and so selection bias could not be avoided. More accurate findings could be observed by using larger sample sizes and by taking blood samples during the first few hours after a stroke to determine whether H19 SNP affects H19 expression and whether NIHSS has a significant correlation with H19 expression level. Although the current study indicates that circulating H19 can be a diagnostic biomarker for IS, further research is required to find reliable blood biomarkers for clinical use.

## Conclusion

This study demonstrated that the circulating H19 in the first 24 h after a stroke might be a diagnostic biomarker for IS. Also, rs217727 polymorphism in the co-dominant and recessive models could be significantly associated with IS risk among the Iranian population. H19 expression was associated with TOAST subtypes in IS patients. However, further large-scale studies are required to confirm our findings and validate the clinical application of this biomarker for IS diagnosis.

## Data Availability

The datasets used and/or analyzed during the current study are available from the corresponding author on reasonable request.

## Abbreviations

AUC: Area under the curve
BMI: Body mass index
CE: Cardio embolism
CI: Confidence interval
EDTA: Ethylene diamine tetra acetic acid
HWE: Hardy-Weinberg equilibrium
IS: Ischemic stroke
LAA: Large-artery atherosclerosis
LncRNA: Long non coding- RNA
NIHSS: National institutes of health stroke scale
OGD/R: Oxygen-glucose deprivation and reperfusion
OR: Odds ratio
ROC: Receiver operating characteristic
ROSIER: Recognition of stroke in the emergency room
Se: Sensitivity
SD: Standard deviation
Sp: Specificity
SVO: Small vessel occlusion
T-ARMS-PCR: Tetra-amplification refractory mutation system-polymerase chain reaction
TBP: TATA-binding protein
UD: Undetermined etiology
